# Potential of recombinant inorganic pyrophosphatase antigen as a new vaccine candidate against *Baylisascaris schroederi* in mice

**DOI:** 10.1186/1297-9716-44-90

**Published:** 2013-10-03

**Authors:** Yue Xie, Sijie Chen, Yubo Yan, Zhihe Zhang, Desheng Li, Hua Yu, Chengdong Wang, Xiang Nong, Xuan Zhou, Xiaobin Gu, Shuxian Wang, Xuerong Peng, Guangyou Yang

**Affiliations:** 1 Department of Parasitology, College of Veterinary Medicine, Sichuan Agricultural University, Ya’an 625014, China; 2 Sichuan Entry-Exit Inspection and Quarantine Bureau, Chengdu 610041, China; 3 The Sichuan Key Laboratory for Conservation Biology on Endangered Wildlife-Developing toward a State Key Laboratory for China, Chengdu Research Base of Giant Panda Breeding, Chengdu, Sichuan 610081, China; 4 China Conservation and Research Center for Giant Panda, Wolong 623006, China; 5 Department of Chemistry, College of Life and Basic Science, Sichuan Agricultural University, Ya’an 625014, China

## Abstract

The intestinal nematode *Baylisascaris schroederi* is an important cause of death for wild and captive giant pandas. Inorganic pyrophosphatases (PPases) are critical for development and molting in nematode parasites and represent potential targets for vaccination. Here, a new PPase homologue, *Bsc*-PYP-1, from *B. schroederi* was identified and characterized, and its potential as a vaccine candidate was evaluated in a mouse challenge model. Sequence alignment of PPases from nematode parasites and other organisms show that *Bsc*-PYP-1 is a nematode-specific member of the family I soluble PPases. Immunohistochemistry revealed strong localization of native *Bsc*-PYP-1 to the body wall, gut epithelium, ovary and uterus of adult female worms. Additionally, *Bsc*-PYP-1 homologues were found in roundworms infecting humans (*Ascaris lumbricoides*), swine (*Ascaris suum*) and dogs (*Toxocara canis*). In two vaccine trials, recombinant *Bsc*-PYP-1 (r*Bsc*-PYP-1) formulated with Freund complete adjuvant induced significantly high antigen-specific immunoglobulin (Ig)G but no IgE or IgM responses. Analysis of IgG-subclass profiles revealed a greater increase of IgG1 than IgG2a. Splenocytes from r*Bsc*-PYP-1/FCA-immunized mice secreted low levels of T helper (Th)1-type cytokines, interferon-γ and interleukin (IL)-2, while producing significantly high levels of IL-10 and significantly elevated levels of IL-4 (Th2 cytokines) after stimulation with r*Bsc*-PYP-1 in vitro. Finally, vaccinated mice had 69.02–71.15% reductions (in 2 experiments) in larval recovery 7 days post-challenge (dpc) and 80% survival at 80 dpc. These results suggest that Th2-mediated immunity elicited by r*Bsc*-PYP-1 provides protection against *B. schroederi*, and the findings should contribute to further development of *Bsc*-PYP-1 as a candidate vaccine against baylisascariasis.

## Introduction

Baylisascariasis is a neglected zoonotic helminthic disease caused by parasitic nematodes of the genus *Baylisascaris* (Nematoda: Ascaridida) with great medical and veterinary significance worldwide [1-3]. The causative pathogens, *Baylisascaris* spp., are widely distributed in the giant panda (*Ailuropoda melanoleuca*), red panda (*Ailurus fulgens*), raccoon (*Procyon lotor*), Ursid species (*Ursus maritimus*, *Ursus arctos pruinosus*, *Selenartos thibetanus mupinensis* and *Ursus arctos lasiotus*) and other mammals including humans and can lead to severe clinical visceral (VLM), ocular (OLM) and neural larva (NLM) migrans in these definitive or intermediate hosts [4-7]. Among them, *Baylisascaris schroederi* is the only endoparasite that appears to be consistently found in the giant panda, a flagship species for wildlife conservation in China, and represents a significant threat to both wild and captive populations [8,9]. In nature, *B. schroederi* infection rates among wild pandas may reach between 50–100%, making it one of the leading causes of death from primary and secondary infection in wild populations [4,10,11]. Zhang et al. demonstrated that the probability of death of wild pandas caused by this pathogen increased significantly between 1971 and 2005, and the associated VLM was the most important cause of death during the recent period 2001–2005 [8]. As with all ascarid species, *B. schroederi* infection follows a trophic pathway by ingestion with life cycle completion without intermediate hosts. This parasite (at the adult stage) usually inhabits the intestines of the giant panda, while its migrating larvae may disseminate into various body tissues. *B. schroederi* can induce extensive inflammation and scarring of the intestinal wall and parenchyma of the liver and lung (mainly caused by larvae), as well as intestinal obstruction, inflammation and even death (caused by adults) in giant pandas [9,12-14]. Until now the control of *B. schroederi* infection in pandas is relied chiefly on chemotherapy, and treatment with antiparasitic drugs requires multiple doses until the animal ceases to expel worms or shed eggs in feces [9]. However, alternative preventative and treatment strategies are needed due to the rapid emergence of multi-drug-resistant ascarids and pollution of the food chain and the environment from chemotherapy, as well as the persistent exposure of host animals to parasites of different stages in their surroundings. Vaccines, particularly target antigens that play crucial roles in the survival, development and reproduction of parasitic nematodes would be an ideal control strategy.

Inorganic pyrophosphatases (PPases, EC 3.6.1.1), a class of cytosolic enzymes catalyzing the hydrolysis of inorganic pyrophosphate (PP_i_) to *ortho*-phosphate (P_i_), are widely distributed among living cells and function in energy metabolism, lipid metabolism and some biosynthetic reactions [15]. PPases are essential for the growth and development of prokaryotes, fungus, nematodes and plants (e.g., tobacco and potato) [16-22]. For parasites, such as *Ascaris* roundworms, PPases are believed to be further involved in molting, as indicated in *Ascaris suum* by RNA-mediated interference and enzyme activity inhibition assays [23,24]. Considering the important roles of PPases in life processes of various organisms including parasitic nematodes, some of these enzymes have been selectively targeted for pharmaceutical and vaccine purposes [25]. For example, an adjuvanted recombinant PPase antigen from ascarids was recently demonstrated to induce a high level of protection (>70%) against *A. suum* challenge in mice, and its potential for use as a candidate vaccine against ascariasis is further suggested in pigs [23]. However, no information on PPases of *B. schroederi* is available to date. More importantly, *B. schroederi*-specific protein antigens as potential targets for vaccines and/or chemotherapeutic agents are still scarce with only three antigen molecules (Bs-Ag1, Bs-Ag2 and Bs-Ag3) available [12,13,26], and the precise mechanism of protective immunity against *B. schroederi* infection has not been determined, although preliminary results have been published on other gastrointestinal parasitic infections showing induction of strong T helper (Th) 2-biased responses in various experimental animal models [27-30].

Therefore, the aims of the present study were (i) to clone and express a new PPase, *Bsc*-PYP-1, from *B. schroederi*; (ii) to investigate the localization of this native protein in adult parasites and its expression profiles at various developmental stages of *B. schroederi*, including embryonated eggs, 2nd and 3rd-stage larvae (L2-L3) and adults; (iii) to test the immunogenicity and protective potential of recombinant *Bsc*-PYP-1 (r*Bsc*-PYP-1) following vaccination with Freund complete adjuvant (FCA) in challenged mice; and finally (iv) to determine the T helper (Th)1 and/or Th2 immune profile induced by vaccination with this recombinant protein based on levels of serum immunoglobulin (Ig)G, its subclasses (IgG1 and IgG2a) and cytokines (e.g., interleukin (IL)-2, IL-4, IL-10 and interferon (IFN)-γ). To our knowledge, this is the first report that evaluates the protective efficacy and corresponding immune mechanisms of the r*Bsc*-PYP-1 protein as a vaccine in a mouse challenge model. The results will contribute to improve our understanding of *Bsc*-PYP-1 as a candidate vaccine against baylisascariasis.

## Materials and methods

### Parasites

*B. schroederi* female adults derived from naturally infected giant pandas were provided by the Department of Parasitology, College of Veterinary Medicine, Sichuan Agricultural University (Ya’an, China). Unembryonated and embryonated eggs were obtained essentially as described elsewhere [31]. *B. schroederi* infective L2 from embryonated eggs and liver-stage L3 from infected mice were collected as previously described [32]. Adult *Ascaris lumbricoides* worms were obtained from patients after treatment with piperazine in Jianyang, Sichuan Province of China. Adult *A. suum* and adult *Toxocara canis* were isolated from infected pigs at a local slaughterhouse in Ya’an and an infected dog in Chengdu, China, respectively. Protein concentrations of phosphate-buffered saline (PBS)-soluble parasite antigens were measured using the micro-bicinchoninic acid (BCA) protein assay kit (Pierce/Thermo Fisher Scientific, Asheville, NC, USA).

### Animals

Six- to eight-week-old female specific-pathogen-free (SPF) BALB/c mice were purchased from the Laboratory Animal Center of Sichuan University (Chengdu, China). New Zealand white rabbits were obtained from the Laboratory Animal Center of Sichuan Agricultural University. All animals were housed under a barrier environment in sterile cages in the laboratory animal house of the National Institute of Animal Health (NIAH) and were fed pelleted food and sterilized water *ad libitum*. Animals were acclimated to these conditions for 1 week prior to the experiment. Institutional Ethical and Animal Care guidelines were followed during the sampling exercise, and all procedures were reviewed and approved by the Institute of Animal Health Animal Care and Use Committee of Sichuan Agricultural University of China.

### Amplification and bioinformatic analysis of *Bsc*-PYP-1 coding sequence

Total RNA isolation from female adults of *B. schroederi* and the first-strand cDNA synthesis were performed according to standard protocols [12]. The resulting cDNA was used as the template for PCR amplification with a sense primer (5′-TAAAGATGGCATTGGCCGCATCG-3′) and an antisense primer (5′-CACTCTTTGATGAAATGCCATCTGTCA-3′) designed to target the *A. suum* AdR44 cDNA sequence (GenBank accession: AB091401). The PCR amplified product was gel-purified, cloned into the pMD19-T vector (TaKaRa, China) and sequenced. An Open Reading Frame Finder [33] and the Lasergene software package for Windows (DNASTAR, Madison, WI, USA) were used to analyze the open reading frame (ORF) of the nucleotide sequence and deduce the amino acid sequences. Similarity comparisons with previously reported sequences in GenBank were performed using DNAMAN version 3.0 (Lynnon Biosoft, Quebec, Canada) and on-line Blast tools at the National Center for Biotechnology Information (NCBI) website [34]. Based on their similarities, multiple sequence alignment and phylogenetic analysis were obtained. Sequences were aligned with ClustalW2 [35], and the phylogenetic tree was constructed by the neighbor-joining (NJ) method [36] and plotted with MEGA 3.1 [37]. In addition, the molecular weight (MW) and isoelectric point (pI) of *Bsc-*PYP-1 were calculated using ProtParam [38], and the signal sequence was predicted with the SignalP 3.0 server [39].

### Expression and purification of recombinant *Bsc*-PYP-1 fusion protein

A partial coding region of *Bsc*-PYP-1 cDNA, except for the predicted signal peptide, was amplified by PCR using a sense primer (5′-CCC*AAGCTT*CGACAATCTCGCAGT-3′) containing a *Hind*III site (italics) and an antisense primer (5′-CCG*CTCGAG*TCACTCTTTGATGAAATGCCATCTGTC-3′) containing an *Xho*I site (italics). The PCR products were digested with *Hind*III and *Xho*I (TaKaRa), gel-purified and ligated into the plasmid expression vector pET32a (+) (Novagen, Madison, WI, USA). The resulting plasmid with a correct *Bsc*-PYP-1 insert was transformed into *E. coli* BL21 (DE3) cells (Invitrogen, Carlsbad, CA, USA) and subsequently grown at 37 °C to an OD_600_ of 0.6 in Luria-Bertani (LB) broth supplemented with 50 μg/mL ampicillin. The transformed cells were induced by adding 1 mM isopropyl-β-D-thiogalactopyranoside (IPTG) for 5 h at 37 °C. The cells were harvested and resuspended in lysis buffer [50 mM NaH_2_PO_4_ (pH 8.0), 10 mM Tris–HCl (pH 8.0), 100 mM NaCl]. The samples were then sonicated until they were no longer viscous. Cell lysates and inclusion bodies were pelleted by centrifugation at 25 000 × *g* for 15 min at 4 °C. The pellets were resuspended in lysis buffer plus 8 M urea and incubated on ice for 1–2 h to completely solubilize the protein. His_6_-tagged r*Bsc*-PYP-1 proteins were expressed in an inclusion body form and purified by Ni^2+^ affinity chromatography using a 10 mL His-Bind Resin column (Novagen) under denaturing conditions as described in the manufacturer’s protocol. Proteins eluted with imidazole were concentrated with Amicon Ultra Centrifugal Filter Devices (Millipore, Billerica, MA) and then dialyzed against PBS, containing decreasing concentrations of urea (8, 6, 4, 3, 2, and 1 M, and PBS only). Thereafter, concentrations of purified proteins were measured with the micro-BCA protein assay reagent (Pierce/Thermo Fisher Scientific), and the potential endotoxin contamination was accessed using the limulus amoebocyte lysate-based gel-clot assay as described elsewhere [40].

### Sera

Rabbit immune serum against *B. schroederi* was obtained as previously described [12]. To obtain mouse polyclonal sera against r*Bsc*-PYP-1, 10 female BALB/c mice were immunized with a subcutaneous injection of 50 μg of r*Bsc*-PYP-1 purified as described above and mixed with FCA (Sigma, St. Louis, USA), followed by 2 booster immunizations (2 weeks apart) using the same route and dose in the same adjuvant. Mice were bled 2 weeks after the second booster immunization. The antisera from the mice were mixed and stored at −20 °C until use.

### Immunoblot and immunohistochemical analyses

For immunoblot analysis, parasite antigens or r*Bsc*-PYP-1 proteins were lysed in an electrophoresis sample buffer, run on 10% SDS-PAGE, transferred onto nitrocellulose membranes and blocked with 5% skim milk in Tris-buffered saline (TBS) buffer for 1 h. Parasite-derived *Bsc*-PYP-1 was detected by incubation of the membranes with the mouse anti-r*Bsc*-PYP-1 serum. To determine the antigenicity of r*Bsc*-PYP-1, rabbit or mouse sera from animals repeatedly inoculated with *B. schroederi* embryonated infective eggs, anti-r*Bsc*-PYP-1 mice serum and naïve rabbit or mouse sera were used. After three washes with TBS-Tween 20 (TBST), the membranes were further incubated for 2 h with 1:200 diluted alkaline phosphatase-conjugated goat anti-mouse or anti-rabbit IgG (ICN Pharmaceuticals, Costa Mesa, CA). Protein signals were visualized using nitroblue tetrazolium and 5-bromo-4-chloro-3-in-dolylphosphate (NBT/BCIP; USB, Cleveland, OH). For immunohistochemical analysis, adult female *B. schroederi* sections were probed with specific anti-r*Bsc*-PYP-1 mouse serum (1:100), followed by a peroxidase-labeled anti-mouse IgG goat antibody (Biosynthesis Biotechnology Co., Ltd., Beijing, China) [19]. Slides were mounted and examined under a light microscope (Nikon Optiphot II, Nikon, Japan).

### Immunization and challenge

For immunization, r*Bsc*-PYP-1 proteins were suspended in PBS (0.01 M, pH 7.4) at a concentration of 600 μg/mL and mixed with an equal volume of FCA (Sigma) as described previously [13]. Control FCA mixed with PBS and control PBS were also used for challenge studies. In the first trial, 90 mice were randomly assigned into three groups (30 animals per group) and injected subcutaneously with the r*Bsc*-PYP-1 suspension, FCA mixed with PBS or PBS alone (100 μL per animal). First and second booster injections were prepared in the same manner and administered at the same dose at 14-day intervals. Two weeks after the final injection, 10 mice from each group were sacrificed, and the spleens were removed aseptically for cytokine assays. The remaining 20 animals in each group were inoculated orally with 3200 *B. schroederi* infective embryonated eggs. Ten out of twenty mice challenged with *B. schroederi* from each group were sacrificed and dissected at one week post-challenge (wpc). After histopathological examination, lungs and livers were removed from the animals and minced with a surgical knife, and larvae were recovered by the Baermann method as described by Slotved et al. [41] and counted under a light microscope (Nikon). For the last 10 remaining mice in each group, mortality was monitored over a period of 80 days after the challenge, and relative percent of survival (RPS) was calculated as follows: RPS = {1 − (% mortality in immunized mice / % mortality in control mice)} × 100, as described elsewhere [42].

In order to further assess the potential efficacy of r*Bsc*-PYP-1 as a new vaccine candidate against *B. schroederi*, the vaccination trial with mice was repeated in trial II essentially as described in trial I, with the exception that the challenge occurred 1 week after the final booster immunization. The overall experimental design is summarized in Table 1. Serum samples in both trials were collected from the tail vein after each immunization and at 42, 49, 63, 77, 84 and 91 days post-vaccination (dpv) for antibody assays and for analysis of the kinetics of humoral immune response throughout the challenge as previously described [12,13,26,43].

**Table 1:** Experimental design of mouse vaccination and challenge

**Trial and experimental group**	**Number of mice**	**0, 2, 4 wpv **^**b**^	**6-wpv **^**b**^		**1-wpc **^**c**^	**1-11 wpc **^**c **^**(80-dpc)**
		**Vaccination**	**Cytokine assay**	**Challenge**	**Larval recovery**	**Mortality**
**(Trial I)**		**(3 times per group)**	**(No. **^**a**^**)**	**(No. **^**a, d**^**)**	**(No. **^**a**^**)**	**(No. **^**a**^**)**
r*Bsc*-PYP-1/FCA/PBS	30	r*Bsc*-PYP-1 (FCA plus PBS)	10	20	10	10
FCA/PBS	30	FCA plus PBS	10	20	10	10
PBS	30	PBS	10	20	10	10
**Trial and experimental group**	**Number of mice**	**0, 2, 4 wpv **^**b**^	**5-wpv **^**b**^	**6-wpv **^**b**^	**1-wpc **^**c**^	**1-12 wpc **^**c **^**(80-dpc)**
		**Vaccination**	**Challenge**	**Cytokine assay**	**Larval recovery**	**Mortality**
**(Trial II)**		**(3 times per group)**	**(No. **^**a**^**)**	**(No. **^**a, e**^**)**	**(No. **^**a, f**^**)**	**(No. **^**a**^**)**
r*Bsc*-PYP-1/FCA/PBS	30	r*Bsc*-PYP-1 (FCA plus PBS)	20	10	10	-
FCA/PBS	30	FCA plus PBS	20	10	10	-
PBS	30	PBS	20	10	10	-

^a^ Number of mice involved in this assay.

^b^ Week(s) post-vaccination.

^c^ Week(s) post-challenge.

^d^ Number of remaining mice in each experimental group after “Cytokine assay” and used for the subsequent determinations of “Larval recovery” and “Mortality”.

^e^ Number of remaining mice in each experimental group after “Challenge”.

^f^ Number of mice derived from “Challenge”.

### Antibody assays

Measurements of *Bsc*-PYP-1-specific serum IgM, IgE, IgG, and IgG-subclass (e.g., IgG1 and IgG2a) antibodies in immunized mice derived from trials I and II were performed using enzyme-linked immunosorbent assays (ELISA). The IgM, IgG and IgG subclass antibody levels were determined with horseradish peroxidase (HRP)-conjugated goat anti-mouse IgM, IgG and IgG subclass antibodies (Bethyl Laboratories, Montgomery, TX, USA). Affinity purified goat anti-mouse IgM, IgG, IgG1 and IgG2a (Bethyl Laboratories) were used as standards. For r*Bsc*-PYP-1-specific IgE measurements, anti-mouse IgE was used as the capture antibody, and IgE was quantified by using biotinylated anti-mouse IgE (Bethyl Laboratories) coupled with the standard produced from rat monoclonal anti-mouse IgE antibody (diluted 1:10 000) (American Research Products, Belmont, MS, USA) as described elsewhere [23,44].

ELISA were performed in polystyrene 96-well micro-titer plates (Invitrogen) using 100 μL reaction mixtures with r*Bsc*-PYP-1 antigen coated at a concentration of 2 μg/mL in 0.1 M carbonate buffer (pH 9.6) as described previously [13]. The plates were washed after incubation of the plates at 4 °C for 14–16 h (and after each subsequent incubation) three times with PBS containing 0.05% Tween 20 (PBS-T). Wells were blocked with 100 μL of PBS-2% bovine serum albumin (BSA) (Sigma) for 2 h at 37 °C, and then incubated with serial two-fold dilutions (100 μL) of test serum samples at 37 °C for 1 h. HRP-conjugated goat anti-mouse IgM (diluted 1:5000), IgG (diluted 1:500) or IgG subclass (diluted 1:5000) antibodies and HRP-conjugated goat anti-rat IgG (diluted 1:1000) (Bethyl Laboratories) antibody were added to the wells in 100 μL for a further incubation at 37 °C for 1 h. Both test sera and conjugates were diluted in PBS. Antibody binding was detected at 37 °C with 100 μL of o-phenylenediamine dihydrochloride (OPD; Sigma) substrate (0.4 mg/mL OPD, 50 mM dibasic sodium phosphate, 25 mM citric acid and 30% H_2_O_2_), and the color reaction was terminated with 100 μL of 2 M H_2_SO_4_. The results were obtained with a microplate reader (Dynatech MR500, Deckendorf, Germany), and the endpoint titer was defined as the highest dilution of serum at which the optical density at 490 nm (OD_490_) was at least above the doubled blank control (OD_490_ 0.15). Negative and blank controls were included on each plate. Antibody concentrations were calculated using a standard curve generated with reference sera (Bethyl Laboratories).

### Spleen cell culture and cytokine assays

Spleen cell suspensions were prepared essentially as described previously [23,45]. After isolation, splenic cells (4 × 10^6^) were subsequently cultured at 37 °C and 5% CO_2_ in 24-well plates in a final volume of 1.0 mL/well. The cells were stimulated with r*Bsc*-PYP-1 antigen (15 μg/mL) for 72 h, and culture supernatants were collected and stored at −80 °C for subsequent cytokine analysis using a commercially available mouse Ad Litteram ELISA Kit with pre-coated plates (ADL, San Diego, CA, USA) for IL-2, IL-4, IL-10 and IFN-γ. Cytokine concentrations were calculated against standard curves constructed with supernatants containing known amounts of mouse recombinant IL-2, IL-4, IL-10 and IFN-γ. All standards and samples were run in triplicate.

### Statistical analysis

The data were expressed as the means ± standard deviations (SD). Comparisons between experimental groups were performed by one-way ANOVA, LSD, Duncan’s test or Scheffe’s test using SPSS13.0 Data Editor (SPSS Inc., Chicago, IL, USA). *P* values < 0.05 were considered to be significant. All experiments were carried out a minimum of two times with 10 mice per group. Percent reduction was calculated as follows: % reduction = [(average number of larvae recovered from control mice − average number of larvae recovered from immunized mice)/average number of larvae recovered from control mice] × 100, as previously described [12]. In addition, the nucleotide sequence determined in the present study has been deposited in the DDBJ/EMBL/GenBank database under accession number GQ859591.

## Results

### Molecular cloning and identification of *Bsc*-PYP-1

**Figure 1:**
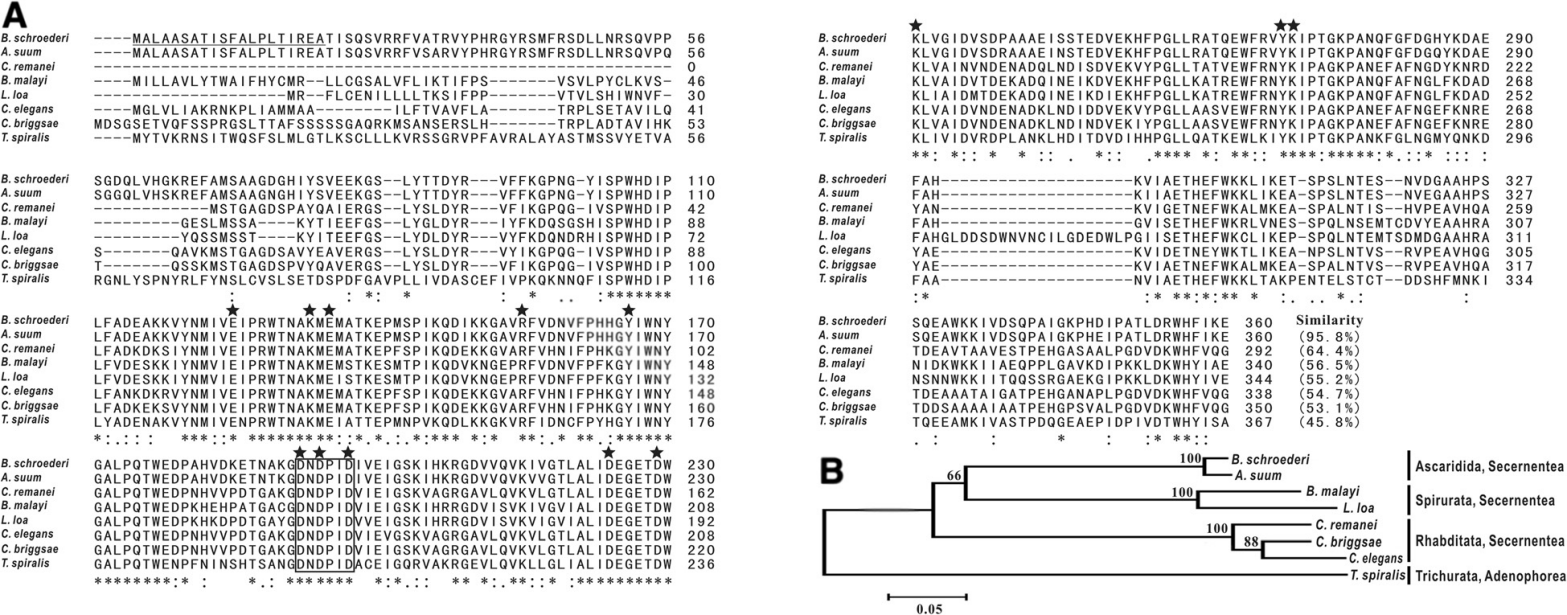
**Sequence alignment and phylogenetic analysis of *Bsc*-PYP-1 with homologous PPases. (A)** Alignment of the deduced amino acid sequence of *Bsc*-PYP-1 with those of homologous PPases from other species. The following sequences were retrieved from the GenBank protein sequence database (accession numbers are indicated in parentheses) and aligned using the ClustalW2 program: *B. schroederi* (GQ859591), *A. suum* (BAC66617), *B. malayi* (EDP36300), *L. loa* (EFO25093), *C. elegans* (NP_001023073), *C. briggsae* (XP_002633752), *C. remanei* (EFP04160) and *T. spiralis* (EFV52164). Regions of identity (*), strong similarity (:) and weak similarity (.) are indicated. Gaps, marked by hyphens, are introduced for better alignment. The putative PPase signature motif is enclosed in the box, and 13 well-conserved residues in all family I soluble PPases are marked with black stars. The predicted signal peptide is underlined. Percentages of sequence similarity with respect to *Bsc*-PYP-1 are shown at the end of each sequence. **(B)** Phylogenetic analysis of the full-length amino acid sequences of *Bsc*-PYP-1 and homologous PPases from the eight nematodes mentioned in **(A)**. The tree was constructed by the NJ method and plotted with MEGA 3.1. Bootstrap values are indicated at the nodes (1000 replications). The scale indicates an estimate of substitutions per site, using the optimized model setting.

The cDNA encoding *Bsc*-PYP-1 with a length of 1,088 bp was obtained by PCR amplification using specific oligonucleotides designed based on the *A. suum* DNA sequence (GenBank ID: AB091401.1). Sequence analysis shows that the cloned cDNA contained a single ORF of 1,083 bp coding for a putative protein of 360 amino acids with a predicted molecular mass of 40.507 kDa and pI of 6.32. The first 20 amino acids corresponded to a signal peptide (Figure 1A). Removal of the signal peptide would result in a mature protein with a molecular mass of 38.478 kDa and a pI of 6.31. Six different clones were sequenced, and no differences were found at the amino acid level.

A homology search for the protein performed using information obtained from NCBI revealed that *Bsc*-PYP-1 shared the highest amino acid sequence similarity with a PPase protein (AdR44) from *A. suum* (95.8%) (GenBank accession: BAC66617), followed by 64.4% similarity with *Cre*-PYP-1 protein from *Caenorhabditis remanei* (GenBank accession: EFP04160), and 45.8–56.5% similarity with putative PPases from parasitic nematodes (*Brugia malayi* (GenBank accession: EDP36300), *Loa loa* (GenBank accession: EFO25093) and *Trichinella spiralis* (GenBank accession: EFV52164)) and free-living nematodes (*Caenorhabditis elegans* (GenBank accession: NP_001023073) and *Caenorhabditis briggsae* (GenBank accession: XP_002633752)) (Figure 1A). Sequence similarities were found throughout the protein but less frequently at both ends. Moreover, a PPase signature sequence (DNDPID) and 13 functionally important and evolutionarily well-conserved active site residues (E:125, K:133, E:135, R:155, Y:170, D:192, D:194, D:197, D:224, D:229, K:231, Y:269 and K:270; in *Bsc*-PYP-1) in family I soluble PPases were also observed in the sequence analysis (Figure 1A). Therefore, based on its similarity and conservation of functionally relevant residues with these other protein members of the PPase superfamily, *Bsc*-PYP-1 was determined to be a family I soluble PPase.

Relationships of the eight nematodes mentioned above, based on the full-length amino acid sequence alignment of the corresponding PPase proteins, were further determined by phylogenic analysis (NJ tree) (Figure 1B). The results were in good agreement with traditional taxonomy, with *B. schroederi* and *A. suum* clustering into a group within the order Ascaridida, *B. malayi* and *L. loa* clustering into another group within the order Spirurata, and *C. remanei*, *C. elegans* and *C. briggsae* grouping into a branch belonging to the order Rhabditata (Figure 1B).

### Expression, purification and biochemical analysis of r*Bsc*-PYP-1

**Figure 2:**
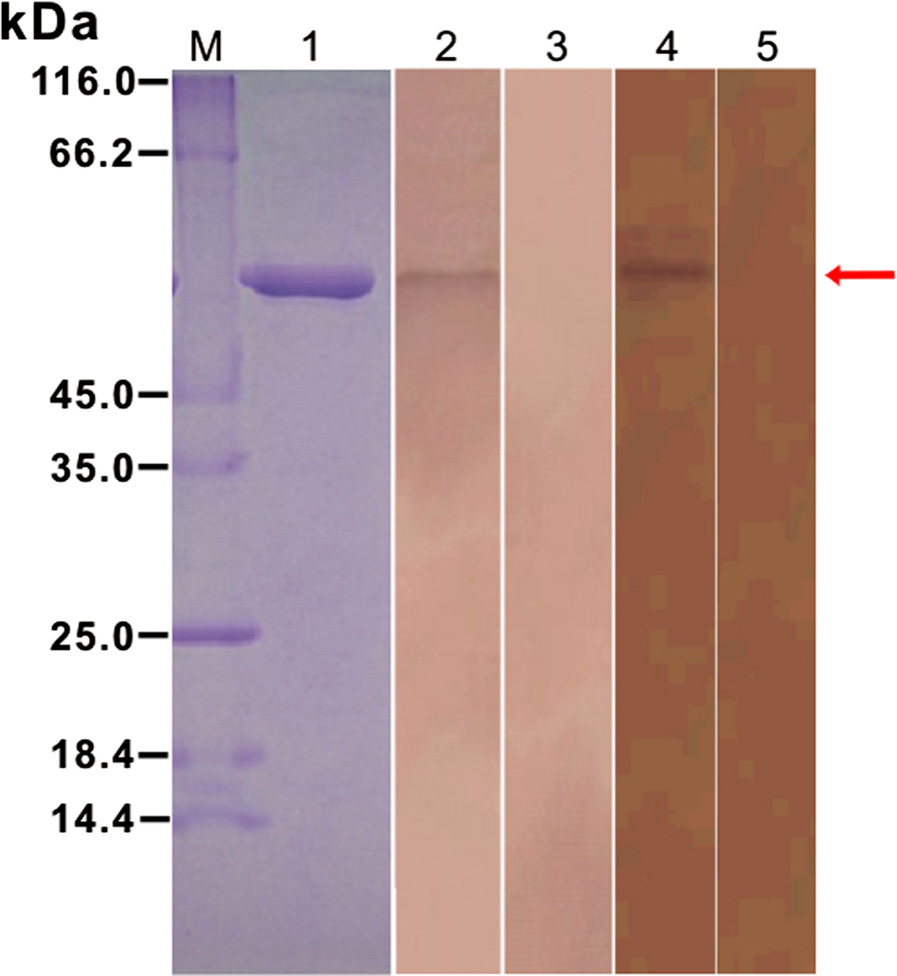
**SDS-PAGE and Western blotting analysis of purified r*Bcs*-PYP-1.** M, molecular mass marker in kDa; lane 1, purified r*Bcs*-PYP-1 after dialysis; lanes 2–5: purified r*Bsc*-PYP-1 was probed with rabbit immune serum against *B. schroederi* (experimental group, lane 2), naive rabbit serum (negative control, lane 3), anti-r*Bsc*-PYP-1 mouse serum (positive control, lane 4) and pre-immune mouse serum (negative control, lane 5), respectively. Two micrograms of protein was loaded in each lane of an SDS-10% polyacrylamide gel, subjected to electrophoresis and blotted onto nitrocellulose membranes. The protein was stained with Coomassie Blue R250 in the gel (lane 1), while the protein bound to serum samples was detected using NBT/BCIP in the Western blot (lanes 2–5). The arrow indicates the location of r*Bsc*-PYP-1-specific bands.

The cDNA fragment encoding mature *Bsc*-PYP-1 was successfully sub-cloned into the pET32a (+) prokaryotic expression vector (Invitrogen), and the recombinant protein was overexpressed in *E. coli* BL21 (DE3) Star cells as a single His_6_-tagged fusion protein with an expected size of ~58 kDa (data not shown). Since the epitope tag fusion peptide in r*Bsc*-PYP-1 was ~20 kDa in size, r*Bsc*-PYP-1 therefore had an approximate molecular mass of 38 kDa, similar to that predicted from the amino acid sequence of *Bsc*-PYP-1. After induction for 5 h with IPTG, the transformed bacterial cells expressing peak amounts of r*Bsc*-PYP-1 were sonicated. r*Bsc*-PYP-1 found mostly in inclusion bodies were made soluble by extraction with 8 M urea (not shown). After purification by affinity chromatography using His binding columns under denaturing conditions, the r*Bsc*-PYP-1 proteins were subsequently refolded by dialysis against PBS containing successively decreasing concentrations of urea. The yield of r*Bsc*-PYP-1 was approximately 4 mg/L of bacterial culture. The purity of the preparation was accessed by SDS-PAGE (Figure 2, lane 1) and further characterized by Western blotting using rabbit immune serum against *B. schroederi* (experimental group), anti-r*Bsc*-PYP-1 mouse serum (positive control), or naïve rabbit and mouse sera (negative controls). A positive band of 58 kDa was observed in the experimental group and positive control, in contrast with the two negative controls, suggesting that r*Bsc*-PYP-1 had good antigenicity (Figure 2, lanes 2–5). The purified r*Bsc*-PYP-1 protein was then used for the production of polyclonal antibodies in mice, immunohistochemical analysis and the examination of immunoreactivity with various immune sera, as well as for vaccine experiments using the *B. schroederi* infection mouse model.

### Identification of the native *Bsc*-PYP-1 antigen in *B. schroederi* and homologues in other ascarids

**Figure 3:**
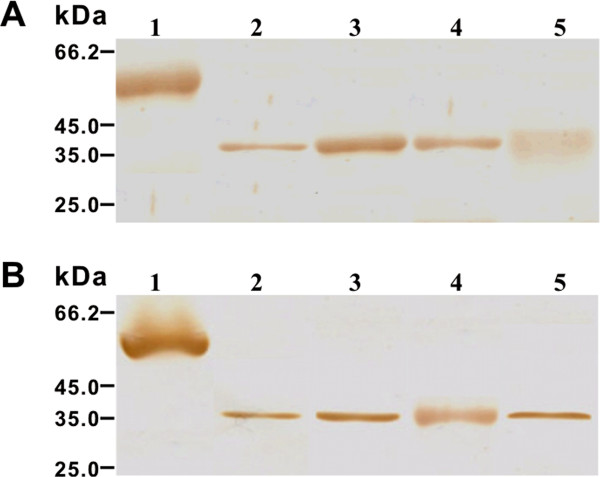
**Identification of endogenous *Bsc*-PYP-1 from *B. schroederi *and homologues from other ascarids. (A)** Identification of endogenous *Bsc*-PYP-1 at various developmental stages of *B. schroederi*. Parasite extracts were prepared essentially as described in Materials and Methods. Eighty micrograms of each parasite extract was separated by SDS-PAGE (10%), and the proteins were then blotted onto a nitrocellulose membrane. Endogenous *Bsc*-PYP-1 proteins bound to mouse anti-r*Bsc*-PYP-1 serum were detected by NBT/BCIP. Lane 1, r*Bsc*-PYP-1 (5 μg); lane 2, female adult worms; lane 3, liver-stage L3; lane 4, L2; lane 5, embryonated eggs. **(B)** Expression of *Bsc*-PYP-1 homologues in ascarid roundworms. Sixty micrograms of protein equivalents of each adult parasite extract were subjected to SDS-PAGE (10%) and then blotted onto a nitrocellulose membrane. *Bsc*-PYP-1 homologues bound to the anti-serum were detected as mentioned above. Lane 1, r*Bsc*-PYP-1 (5 μg); lane 2, *A. lumbricoides*; lane 3, *B. schroederi*; lane 4, *A. suum*; lane 5, *T. canis*.

The native *Bsc*-PYP-1 antigen was identified at various stages of development in *B. schroederi*. Expression of *Bsc*-PYP-1 from parasites was evaluated by immunoblot analysis using extracts prepared from embryonated eggs, L2, liver-stage L3 and female adult worms. Anti-r*Bsc*-PYP-1 mouse serum reacted strongly with a 38-kDa parasite-derived antigen from extracts of all *B. schroederi* life-cycle stages (Figure 3A). By contrast, sera from pre-immune mice did not react with any antigens in the parasite extract (not shown). These findings revealed that the endogenous *Bsc*-PYP-1 protein was commonly expressed in *B. schroederi* at all developmental stages. Additionally, we also performed immunoblot analysis of human (*A. lumbricoides*), swine (*A. suum*) and dog (*T. canis*) roundworms using anti-r*Bsc*-PYP-1 mouse serum. The mouse serum immunoreacted with a 38-kDa PBS-soluble protein from *A. lumbricoides*, the same size as that of parasite-derived *Bsc*-PYP-1. Interestingly, 38-kDa immunoreactive bands were also observed in PBS-soluble extracts from *A. suum* and *T. canis* with nearly equal intensities as parasite-derived *Bsc*-PYP-1 observed in *A. lumbricoides*, indicating the presence of a *Bsc*-PYP-1 homologue in each of these three roundworms (Figure 3B). Sera from mice prior to immunization did not react with any of the antigens present in the parasite extracts (data not shown).

### Immunohistochemical localization of endogenous *Bsc*-PYP-1 in adult female *B. schroederi* worms

**Figure 4:**
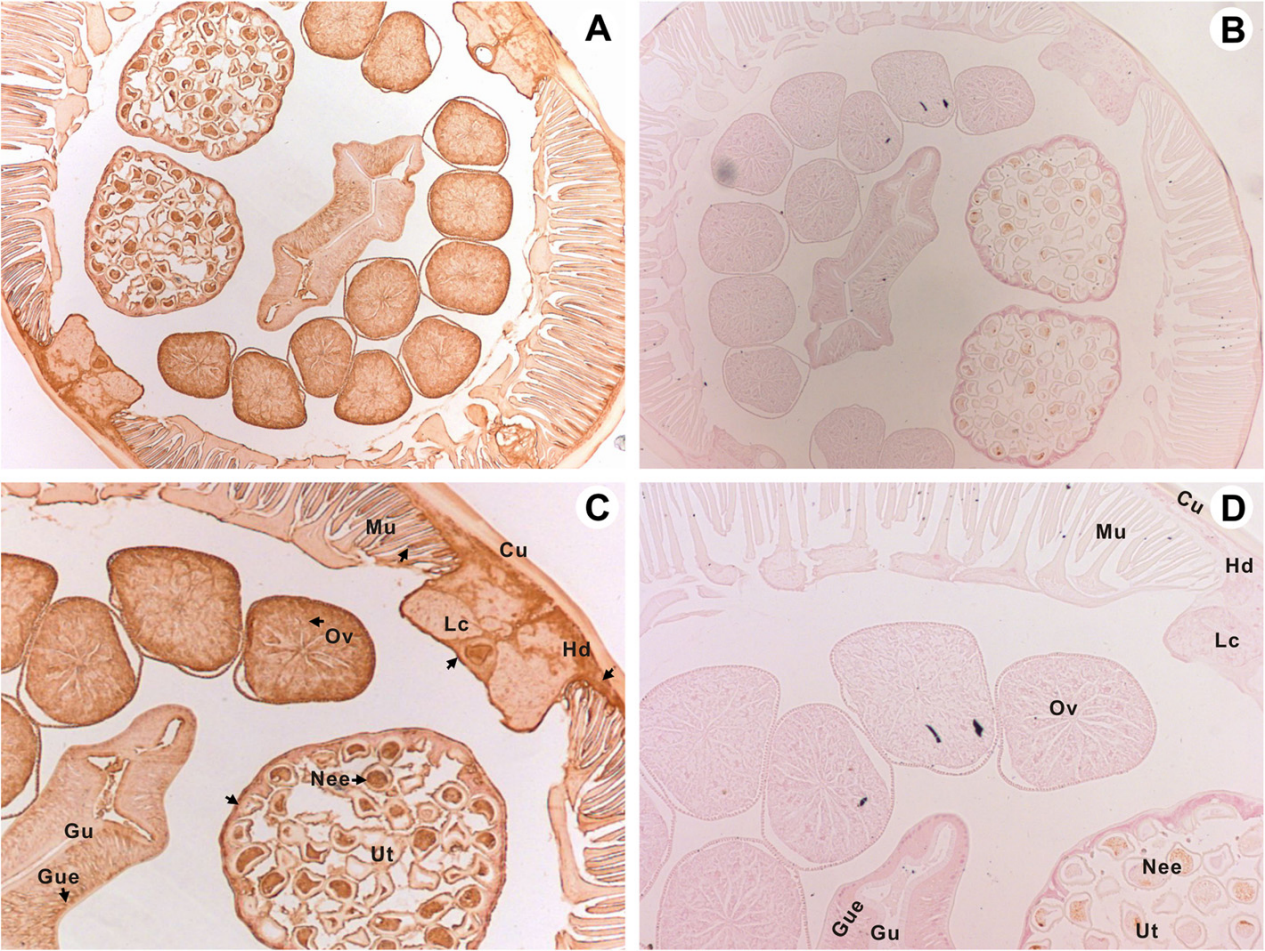
**Immunohistochemical localization of endogenous *Bsc*-PYP-1 in *B. schroederi *female adult worms.** Worms were fixed in paraformaldehyde and embedded in paraffin as described in Materials and Methods. The sections (6-μm thickness) were incubated with either mouse anti-r*Bsc*-PYP-1 serum at 1:100 **(A)** or pre-immune serum at 1:100 **(B)** diluted in PBS. **(C)** and **(D)** (both 20×) are magnified areas of **(A)** and **(B)** (both 10×), respectively. Arrows indicate antibody-labeled regions. Abbreviations: Mu, muscle; Cu, cuticle; Lc, lateral chord; Hd, hypodermis; Ov, ovary; Gu, gut; Gue, gut epithelium; Ut, uterus; Nee, non-embryonated eggs.

The localization of endogenous *Bsc*-PYP-1 protein was determined by immunohistochemistry using anti-r*Bsc*-PYP-1 mouse serum and naïve mouse serum. Specific staining was clearly observed in sections probed with *Bsc*-PYP-1-specific serum but not in those probed with normal mouse serum (Figures 4A-D). The results show that endogenous *Bsc*-PYP-1 proteins were localized in various tissues, such as the hypodermis, dorsal and lateral hypodermal chord, muscle tissues, gut epithelium, non-embryonated eggs within the uterus, uterus and ovary of a female adult *B. schroderi* (Figures 4A and 4C). Moreover, the ubiquitous presence of *Bsc*-PYP-1 homologous proteins in various organs of ascarids from humans and swine was also detected (data not shown), consistent with observations from previous studies [19].

### Reactivity of r*Bsc*-PYP-1 with various immune sera

The immunoreactivity of r*Bsc*-PYP-1 with sera from rabbits and mice repeatedly inoculated with *B. schroderi* infective embryonated eggs was examined by immunoblot analysis. Both sera reacted with the r*Bsc*-PYP-1 band with equal intensities, further demonstrating the good antigenicity and immunogenicity of the recombinant protein (Figure 2 and data not shown). Meanwhile, rabbit and mouse pre-immune sera did not react with r*Bsc*-PYP-1.

### Immunoprotective effect of r*Bsc*-PYP-1 as a recombinant protein vaccine against *B. schroederi*

**Figure 5:**
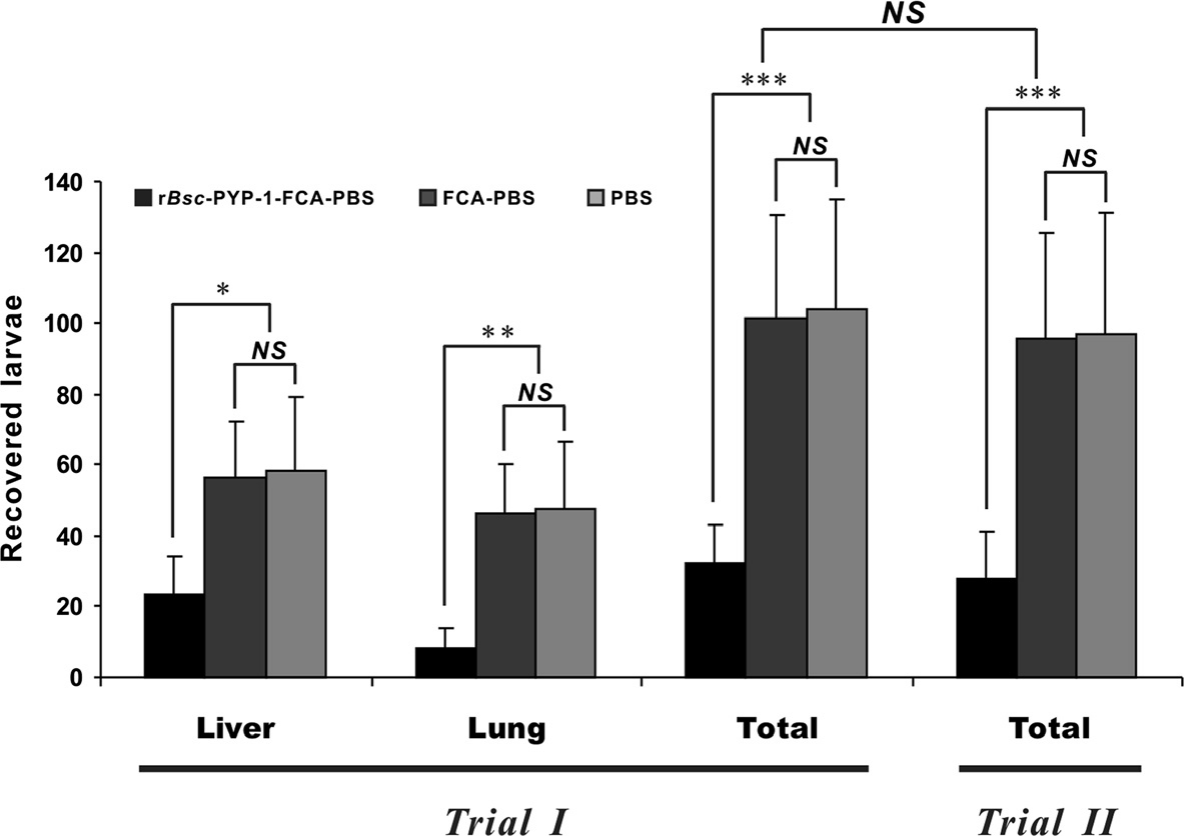
**Numbers of *B. schroederi *larvae recovered from livers, lungs or both in mice vaccinated with r*Bsc*-PYP-1 coupled with FCA.** Mice were vaccinated three times subcutaneously and then inoculated orally with 3200 *B. schroederi* infective embryonated eggs at 1 week (trial II) or 2 weeks (trial I) after the final vaccination, as described in Materials and Methods. Mice were sacrificed at 1 wpc, and the migrating larvae were recovered from livers or lungs. The results are expressed as the mean ± SD in each group of 10 mice. Asterisks indicate that the mean value was significantly lower than that of the group vaccinated with PBS plus FCA or PBS alone (*P < 0.05, **P < 0.01, ***P < 0.001). *NS* denotes no statistically significant difference, and the error bars indicate SD.

Efficacy of the r*Bsc*-PYP-1 protein vaccine against *B. schreoderi* migratory-phase infection was investigated in BALB/c mice receiving one primary vaccination and two boosters, followed by an oral challenge with 3200 *B. schreoderi* infective embryonated eggs, based on two independent trials (trials I and II) performed under the same conditions, with the exception of different challenge timepoints, as summarized in Table 1. In trial I, at one wpc, mice vaccinated with r*Bsc*-PYP-1/FCA had a significantly (*P* = 0.024) reduced number of *B. schreoderi* liver-stage L3 recovered from the liver and a further significant (*P* = 0.0085) reduction in recovered larvae from lungs compared with either mice vaccinated with PBS plus FCA or PBS alone, giving a total reduction of 69.02% in the number of recovered larvae from immunized mice compared with the controls (*P* < 0.001) (Figure 5). No significant differences were observed between the two control groups. In trial II, a significant larvae reduction (including in the livers and lungs) was also shown in the group vaccinated with r*Bsc*-PYP-1/FCA (71.15%, *P* < 0.001) (Figure 5). The reduction of parasitic load in trial II was 2.13% greater than that in trial I, but this difference was not statistically significant (*P* > 0.05). Moreover, in both trials, histopathological observations of livers and lungs removed from vaccinated mice or non-vaccinated mice show that the occurrence of typical verminous interstitial hepatitis (“milk spotted liver”) or verminous pneumonia (pulmonary hemorrhage) following challenge infections was notably reduced in the vaccinated group, compared with the control groups (data not shown).

**Figure 6:**
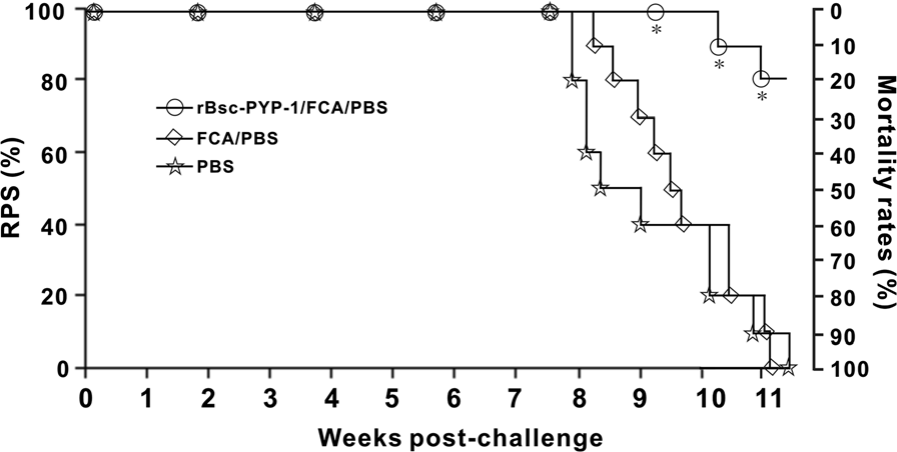
**RPS rates in immunized mice.** Mice were immunized three times with r*Bsc*-PYP-1/FCA/PBS (O), FCA/PBS () or PBS (), followed by oral challenge with 3200 *B. schroederi* infective embryonated eggs at 2 weeks after the last immunization in trial I. Each group consisted of 10 mice. All mice were monitored for mortality for 80 days after the challenge, and the mortality rates (right side) corresponding to RPS rates (left side) are shown. Statistically significant differences (*P* < 0.05) are indicated by asterisks.

To further test whether immunization with r*Bsc*-PYP-1 could also arrest larval development and decrease the risk of death caused by visceral larval migrans, the 10 remaining mice in each group in trial I were monitored for survival. No mortality was observed among all experimental groups until 8 wpc. Thereafter, the cumulative mortalities of the mice vaccinated with adjuvanted r*Bsc*-PYP-1, PBS plus adjuvant and PBS alone were 20%, 100% and 100%, respectively, which corresponded to a relative percent of survival of 80% for r*Bsc*-PYP-1-vaccinated mice compared with the controls (Figure 6). At 80 days after challenge, all surviving mice were sacrificed. *B. schreoderi* was the only parasitic nematode recovered from the liver, lung, kidney, brain, spleen and muscle tissues of moribund and sacrificed mice during this period, suggesting that mortality was caused by *B. schreoderi* infection.

### Serum antibody responses to vaccination in trials I and II

**Figure 7:**
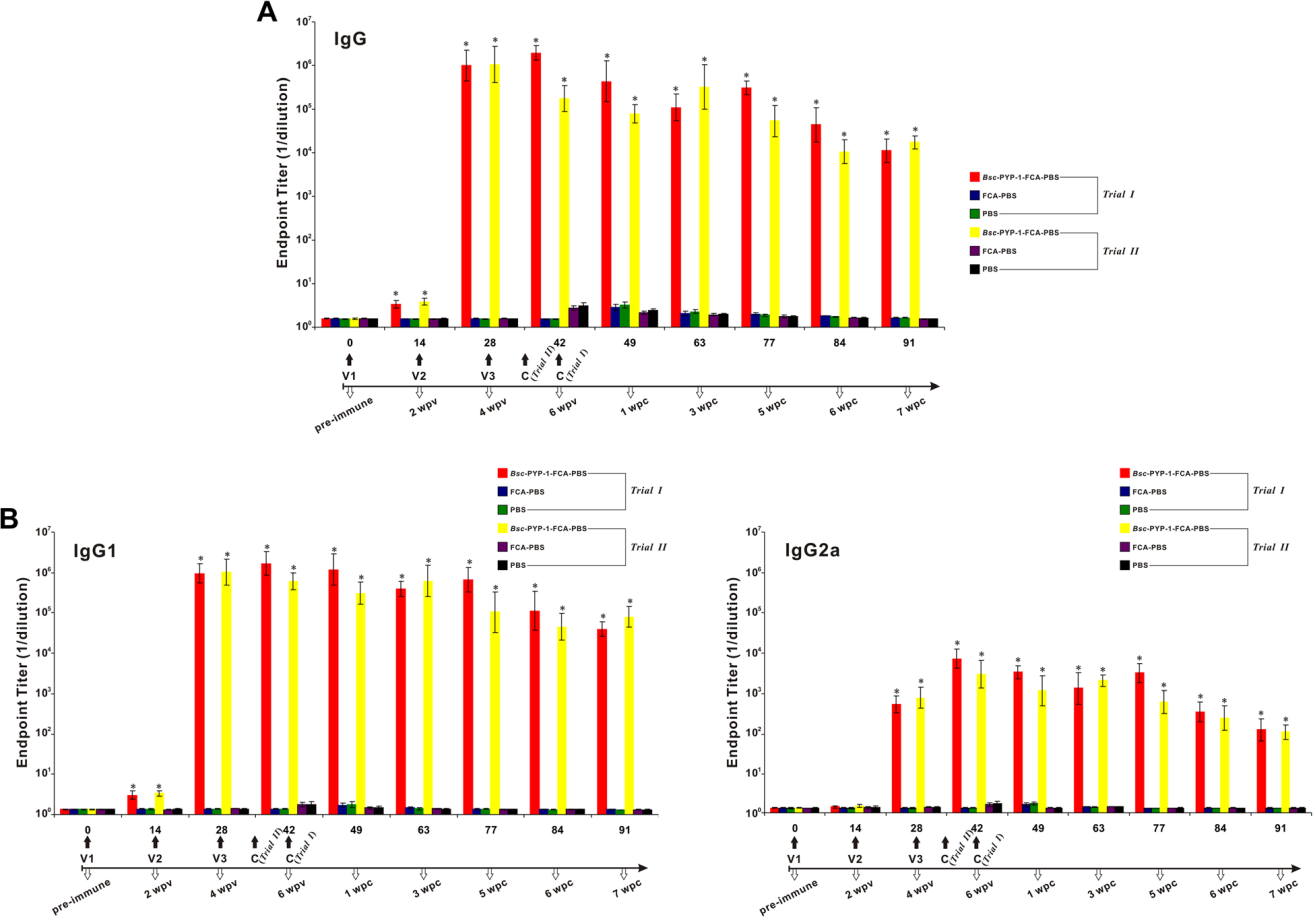
**Serum IgG and IgG subclass (IgG1 and IgG2a) responses induced by r*Bsc*-PYP-1 in mice.** Mice were tested for serum IgG and its subclasses before and after challenge with *B. schroederi* infective embryonated eggs either at 5 wpv (trial II) or at 6 wpv (trial I). Mice were subcutaneously vaccinated three times with r*Bsc*-PYP-1 mixed with FCA in PBS at 14-day intervals, and serum samples were collected from the tail vein after each vaccination and at 0 (challenge or 6 wpv), 1, 3, 5, 6 and 7 wpc. Endpoint titers of anti-r*Bsc*-PYP-1 IgG **(A)** and IgG-subclass **(B)** antibodies from immunized and control mice were determined by ELISA. Values are expressed as the mean ± SD for each group of six mice. Vaccination times (V1, V2, and V3) and challenge (C) are marked with black arrows, and asterisks in **A** and **B** indicate statistically significant differences between the r*Bsc*-PYP-1/FCA/PBS vaccination group and two control groups (PBS plus FCA or PBS alone) (*P* < 0.001). Error bars represent SD.

To evaluate the potential of r*Bsc*-PYP-1 to induce host-protective antibodies, levels of r*Bsc*-PYP-1-specific total IgG, IgE, IgM and IgG-subclasses (including IgG1 and IgG2a) antibodies in vaccinated mice sera derived from trials I and II were measured at various times (0, 2, 4, 6, 7, 9, 11, 12, 13 wpv). As shown in Figure 7A, the amount of r*Bsc*-PYP-1-specific IgG in mice vaccinated with r*Bsc*-PYP-1/FCA within trial I significantly (*P* < 0.001) increased after the first immunization and remained at a high level until the end of the study period, i.e. 91 days, compared with either the adjuvant control or blank control. Interestingly, two peaks of r*Bsc*-PYP-1-specific IgG appeared during this course, with one occurring at 6 wpv and another at 5 wpc (Figure 7A). No detectable antigen-specific IgE response was observed in these mice sera even after the first and second booster vaccinations (data not shown). A similar pattern of antibody induction was also seen for r*Bsc*-PYP-1-specific IgM, although the amount increased only slightly at 1 wpc and lasted 2 weeks at a low level. Interestingly, no significant difference in the r*Bsc*-PYP-1-specific IgM level was found compared with the controls (not shown). To further evaluate the type of immune response (Th1 or Th2) induced by r*Bsc*-PYP-1 in vivo, IgG subclasses were also measured. Both IgG1 and IgG2a levels were significantly (all *P* values < 0.001) increased at 2, 4 and 6 wpv compared with the two controls (PBS plus adjuvant and PBS alone), but vaccination with r*Bsc*-PYP-1 induced a more clear pattern of IgG1 response (Figure 7B).

In trial II, compared with the controls, a significant IgG response was also detected in the group immunized with r*Bsc*-PYP-1/FCA (*P* < 0.001) (Figure 7A); however, due to the challenge timepoint being earlier than that in trial I, the level of r*Bsc*-PYP-1-specific IgG in immunized mice seemed to decline after 4 wpv to lower levels than those in trial I until the end of the study, with the exception of the 3 and 7 wpc timepoints. Similar antibody changes were also observed in the IgG1 and IgG2a subclasses (see Figure 7B). No significant differences in the trend or level of antigen-specific IgE and IgM responses were found in trial II when compared with those in trial I (data not shown). Additionally, as seen in Figure 7, the IgG, IgG1 or IgG2a titers in the two controls of these two trials, appeared to slightly increase after mice were challenged with *B. schreoderi* infective embryonated eggs, although statistically significant differences were still observed between them and the vaccination group. In contrast, no significant responses were noted prior to exposure to this parasite.

### Cytokine responses in stimulated splenocyte culture supernatants

**Figure 8:**
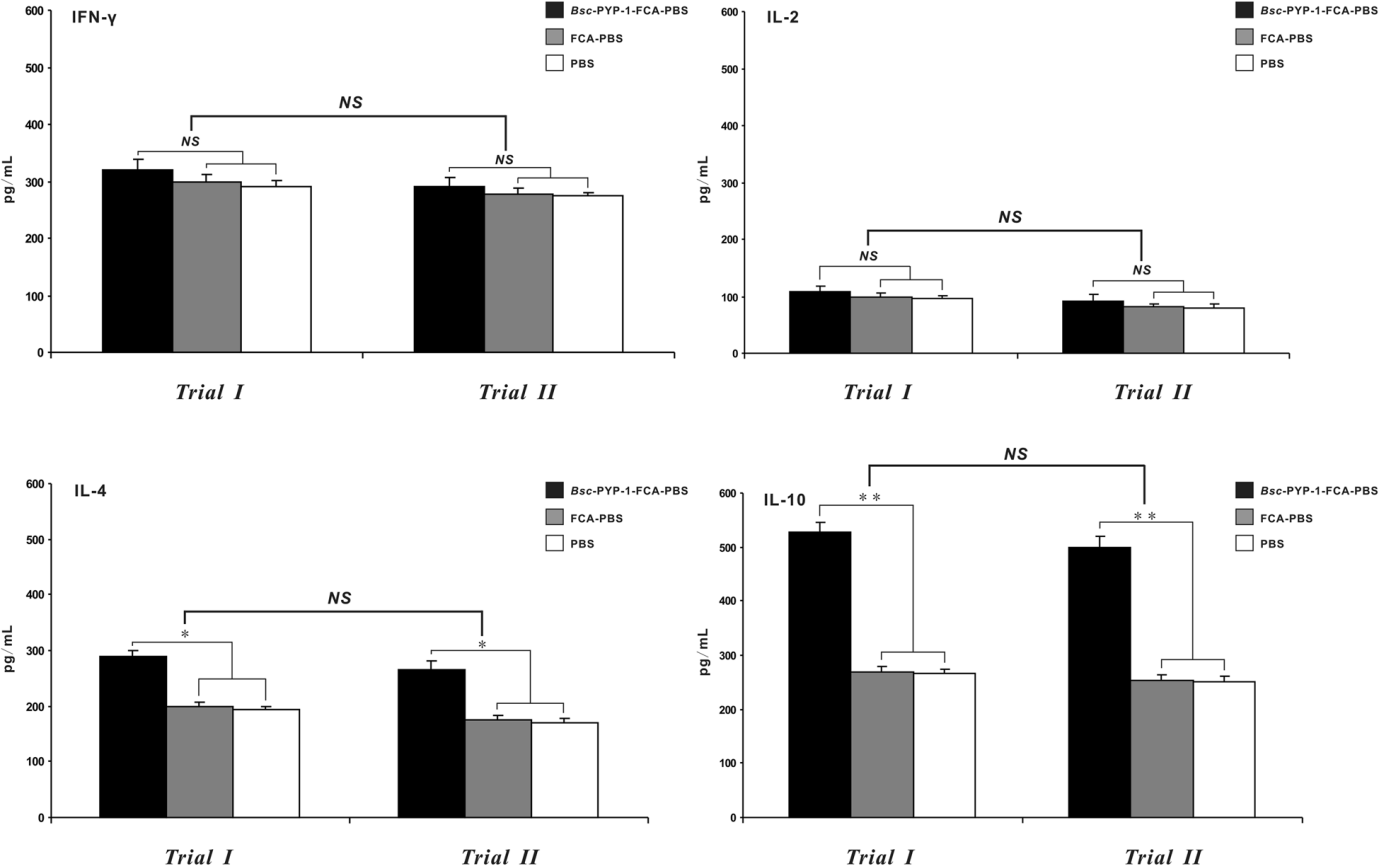
**Levels of cytokines produced by splenic T cells from mice subcutaneously vaccinated with r*Bsc*-PYP-1.** Splenocytes derived from trials I and II were stimulated with r*Bsc*-PYP-1 (15 μg/mL) for 72 h in vitro, and concentrations of cytokines (IFN-γ, IL-2, IL-4 and IL-10) in the supernatant were measured by ELISA. Data are expressed as the mean ± SD for each group of 10 mice. Each sample was examined in triplicate. Asterisks indicate that the mean value was significantly higher than that of the group immunized with PBS plus FCA or PBS alone (*P < 0.01, **P < 0.001). *NS* denotes no statistically significant difference, and error bars indicate SD.

Cytokine secretion in r*Bsc*-PYP-1-stimulated splenocyte culture supernatants was quantified by ELISA in trial I and further confirmed in trial II. In trial I, in vitro-stimulated splenic T cells from mice vaccinated with r*Bsc*-PYP-1/FCA secreted a significantly higher level of IL-10 (*P* < 0.001) and a significantly increased level of IL-4 (*P* < 0.01) (Th2-type cytokines), compared with the levels in stimulated T cells from two non-vaccinated controls (FCA mixed with PBS and PBS alone) (Figure 8). However, stimulated T cells from immunized mice released slight but nonsignificant increases of both IFN-γ and IL-2 (Th1-type cytokines) levels (*P* > 0.05) compared with those from T cells of non-vaccinated controls (Figure 8). Together, these results indicate that the r*Bsc*-PYP-1 antigen induced a predominantly Th2-type immune response in experimentally vaccinated mice. Considering the inherent variability of measuring cytokine responses ex vivo, this assay was repeated in a second trial under the same experimental conditions. Encouragingly, a similar cytokine secretion profile was detected in trial II and further validated the conclusion proposed above. In comparing these results from trials I and II, no statistically significant differences were seen (Figure 8).

## Discussion

*B. schroederi* is increasingly recognized as an important cause of mortality in giant pandas [4,7,8,14], but a preventative vaccine is currently lacking. As PPases are essential for growth and viability in various organisms including parasites [21,22], they are attractive targets for vaccine development against parasitic infections. Specifically, Islam et al. showed in an *A. suum* mouse infection model that vaccination with the *E. coli*-expressed recombinant antigen rAsPPase could confer significant host-protective immunity against parasite challenge [23]. Unfortunately, no information on PPases of *B. schroederi* has been reported thus far. In the present study, a new *B. schroederi* PPase designated as *Bsc*-PYP-1 was identified, cloned and expressed, and its potential as a vaccine for the control of *B. schroederi* infection was evaluated using a mouse challenge model.

Extensive protein database searches revealed that the deduced amino acid sequence of *Bsc*-PYP-1 is highly similar to the *A. suum* PPase protein AdR44 and moderately similar to PPase homologues from other nematode species, while failing to share comparable levels of similarity (all values < 20%) with any available PPase proteins from mammals, including the giant panda, the specific host of *B. schroederi* (Figure 1A). These observations along with the presence of the PPase signature domain and 13 evolutionarily well-conserved residues, suggest that *Bsc*-PYP-1 is a novel nematode-specific PPase with the likely potential to be developed as a vaccine candidate against disease caused by *B. schroederi* in the giant panda. Since parasite-specific molecular antigens with no or low similarity to host proteins are desirable as vaccines for parasitic infections because of antibodies induced against them without cross-reaction with host proteins [46-48]. Similar parasite-specific antigens have been reported in other nematodes, such as human filarial *B. malayi* (ALT1, 2 antigens) [49] and swine roundworm *A. suum* (14 kDa and 16 kDa antigens) [44,50]. By immunoblot analysis, r*Bsc*-PYP-1 shows good antigenicity and immunogenicity, which are properties of an ideal vaccine candidate. Therefore, we further examined whether r*Bsc*-PYP-1 could provide protection in a *B. schroederi* challenge mouse model. Encouragingly, our data from two separate trials consistently show that immunization of BALB/c mice (one primary immunization and two boosters at 2-week intervals) with r*Bsc*-PYP-1 coupled with FCA resulted in protection against migrating *B. schreoderi* larvae in the liver and lung. For both trials I and II, the protection was represented by significant reductions in the number of recovered *B. schreoderi* liver-stage L3 in livers and larvae from lungs after challenge, compared with control mice (Figure 5 and data not shown). Additionally, histopathological damage as revealed by the typical verminous interstitial hepatitis or verminous pneumonia [32] present on the liver and lung of r*Bsc*-PYP-1-vaccinated mice was also significantly reduced following challenge (not shown). Thus, our study indicates that subcutaneous vaccination of mice with r*Bsc*-PYP-1/FCA efficiently induced protective immunity against *B. schroederi* larval infection.

The immunoprotective effects of r*Bsc*-PYP-1 were further confirmed by the high level of protection from death after parasitic challenge in trial I, with an RPS rate of up to 80% in the vaccinated group monitored until 80 dpc, compared with controls (Figure 6). It is possible that vaccination could alter larval migration from the liver to lung to other sites that are less lethal than normal (e.g., diversion from the brain) or inhibit larval molting and development in a manner that is less pathogenic. However, such a high RPS observed in the vaccinated group appeared to be closely associated with specific r*Bsc*-PYP-1 humoral and cellular immune responses. We found that mice immunized with r*Bsc*-PYP-1 produced high antigen-specific IgG antibody titers during the experiment. The anti-r*Bsc*-PYP-1 IgG1 levels increased to a greater extent than that of anti-r*Bsc*-PYP-1 IgG2a, suggesting that r*Bsc*-PYP-1/FCA vaccination induced a Th2-type immune response. Interestingly, no antigen-specific IgE response was detected in these mice, suggesting that r*Bsc*-PYP-1 may be a non-allergenic molecule, and immune responses to r*Bsc*-PYP-1 were not mediated by hypersensitivity reactions. IgM responses were also detected at only a background level until 1 wpc (data not shown). Similar conclusions were drawn in the repeat experiment (trial II) with different challenge timepoints. Previous studies have associated parasite-specific IgG and IgE responses with protective immunity to human ascariasis [51,52]. Similar antibody responses have also been reported in experimental pigs or mice immunized either by repeated inoculation of *A. suum* embryonated eggs or by parasite crude or recombinant antigens [50,53]. However, to date, the functional significance of these two antibody types in protective responses to parasite infection remains to be defined. It is reasonable to assume that protection, as determined by RPS, in the *B. schroederi* infection mouse model may be associated with high levels of total IgG and both IgG subclasses (IgG1 and IgG2a). Further investigation using pooled sera from r*Bsc*-PYP-1/FCA immunized mice for passive immunization may be used to examine the potential of these antibodies in blocking *B. schroederi* larval migration and consequently death in experimentally infected mice. In addition, the antibody titers of total IgG, IgG1 and IgG2a detected in the controls (trials I and II) increased slightly after challenge. This pattern of antibody responses, which is a common phenomenon in vaccine studies of *Baylisascaris* spp. (e.g., *Baylisascaris transfuga*) where the kinetics of tested antibodies (including IgG and IgG subclasses) in non-vaccinated animals characteristically rise after challenge (H.M. Nie and Y. Fu, personal observations and unpublished data), is putatively attributed to low quantities of the native antigen present in larvae and the tissue localization of the endogenous protein in these parasites.

Analysis of antibody responses indicates that r*Bsc*-PYP-1 plus FCA induced a remarkable Th2-type protective immunity in vaccinated mice. This conclusion was supported by subsequent cytokine analyses from two independent trials, in which the in vitro-stimulated splenic T cells from r*Bsc*-PYP-1-immunized mice secreted a significantly high level of IL-10 and a significantly increased level of IL-4 (both Th2-type cytokines), while producing only low levels of the Th1-type cytokines IFN-γ and IL-2. Since IL-10 is well-known to play an antagonist role in the production of IFN-γ and IL-2, it was likely that a high production of IL-10 by CD4^+^ T cells suppressed the synthesis of these cytokines by Th1 cells and CD8^+^ lymphocytes in mice subcutaneously immunized with r*Bsc*-PYP-1/FCA [54,55]. Additionally, an elevated level of IL-4 (*P* < 0.01) in immunized mice apparently did not contribute to IgE production in our study. By contrast, previous investigations in mice exploring regulatory and biological functions of parasite-induced cytokine responses indicated IL-4 as a potent cytokine with the ability to drive IgE production [56]. Thus, further analysis using IL-4-deficient mice is needed to clarify this phenomenon. Islam et al. showed that mice immunized with rAs24 coupled with FCA elicited production of a dominant Th2-type cytokine IL-10, together with IFN-γ but not IL-2, and conferred protection against challenge as demonstrated by a 58% reduction in recovery and stunted development of *A. suum* lung-stage larvae at 7 dpc [57]. Hence, we hypothesize that IL-10 may play an important role in cellular immune responses induced by r*Bsc*-PYP-1/FCA and even may directly contribute to the protective immunity against *B. schroederi* infection in mice. Interestingly, this hypothesis is supported by a previous finding that IL-10 is crucial for resistance and survival of mice infected with *Trichuris muris*[58].

Currently, effector mechanisms that induce stunted development and prevent migration of *B. schroederi* larvae after exiting through the host’s gut wall remain poorly understood. Encouragingly, several studies have verified that specific IgG antibodies from mice immunized with *A. suum* recombinant antigens (rAs24 or rAsPPase) can penetrate the cuticle of *A. suum* larvae during the course of their liver-lung migration and neutralize the corresponding endogenous proteins, thereby interfering with larval migration and/or growth and development [23,57]. Additional studies have shown that the nematode cuticle is a dynamic structure with important absorptive, secretory and enzymatic functions and not merely an inert covering as was once believed [23,59]. These observations together with our analysis of IgG responses (Figure 7A) suggest a conspicuous reduction of r*Bsc*-PYP-1-specific antibody after challenge with infective embryonated eggs, probably due to similar antibody neutralization of endogenous *Bsc*-PYP-1 proteins from migrating *B. schroederi* larvae. This assumption may be tested in future in vitro inhibition or toxicity studies of *B. schroederi* larvae using anti-r*Bsc*-PYP-1 IgG. It should be noted that the protective responses to r*Bsc*-PYP-1 against intestinal adult stage worms were not examined in the present study. Indeed, immune responses against tissue-dwelling or tissue-migratory helminths can be different from those against gastrointestinal parasites [60].

Nematode parasites (including *B. schroederi*) are multicellular eukaryotes with complex life cycle stages, and the adults possess a full complement of immune evasion strategies [61]. Therefore, finding a specific immune mechanism that would effectively decrease worm burden is extremely difficult, and protection often involves both humoral and cell-mediated responses [62,63]. In the present study, immunization with r*Bsc*-PYP-1/FCA induced strong Th2-biased humoral and cellular immune responses and conferred protection against *B. schroederi* challenge, as characterized by significant (*P* < 0.001) levels of antibodies (e.g., IgG and IgG subclasses, IgG1 > IgG2a) and IL-10 cytokine (Figure 7 and Figure 8), as well as significant (*P* < 0.001) reductions (69.2% and 71.15%) in larval recovery (Figure 5) compared with both controls. These results, to a certain extent, implied the potential importance of a r*Bsc*-PYP-1-induced Th2-mediated immune mechanism in fighting *B. schroederi* infection. This current study coupled with the existing evidence from other gastrointestinal nematode infections in various experimental animal models [27-30], confirmed once again that a nematode-specific Th2 response is essential for host parasite clearance during infection. Certainly, more studies should be performed to reinforce or renew this conclusion. Furthermore, the percentage of reduction in larval load caused by *Bsc*-PYP-1 in our study (69.2–71.15%) was comparable or slightly higher than those previously reported with *B. schroederi*-specific antigens: Bs-Ag1 (69.2%) [26], Bs-Ag2 (63.66%) [12] and Bs-Ag3 (62.91%) [13]. To a certain extent, these results suggest that under the same route and dose in the same adjuvant, *Bsc*-PYP-1 may more effectively confer protective immunity against migrating larvae of *B. schroederi* in the host, the giant panda, compared to the three other proteins. Future clinical vaccine examinations simultaneously testing these four *B. schroederi*-derived antigens in pandas would confirm this assertion. Of course, the vaccination protocol for r*Bsc*-PYP-1, as a new candidate antigen screened from the parasite *B. schroederi*, will require further optimization by evaluating different adjuvants and administration routes in order to enhance immune responses.

In summary, we identified and characterized a new *B. schroederi* protective antigen commonly expressed in all life-cycle stages of this parasite as a PPase (*Bsc*-PYP-1). Our results demonstrate that subcutaneous vaccination of mice with *E. coli*-expressed r*Bsc*-PYP-1, coupled with FCA, resulted in a remarkable Th2-type protective immunity against *B. schroederi* challenge, and the protection was evident by the significant reduction of parasitic load and the high survival rate in vaccinated mice. These findings provide insight into r*Bsc*-PYP-1-induced mechanisms that trigger Th2-type immune responses, which may be important in host-protective immunity against *B. schroederi* larvae infection, and they should contribute to further development of *Bsc*-PYP-1 as a candidate vaccine against ascariasis, including baylisascariasis.

## Abbreviations

PPases: Inorganic pyrophosphatases; Bsc-PYP-1: *B. schroederi* PPase; rBsc-PYP-1: Recombinant *Bsc*-PYP-1; FCA: Freund’s complete adjuvant; PBS: Phosphate-buffered saline; PBS-T: PBS containing 0.05% Tween 20; TBS: Tris-buffered saline; TBST: TBS-Tween 20; IPTG: Isopropyl-β-D-thiogalactopyranoside; SDS-PAGE: Sodium dodecyl sulfate-polyacrylamide gel electrophoesis; ELISA: Enzyme-linked immunosorbent assay; BSA: Bovine serum albumin; OPD: O-phenylenediamine dihydrochloride; OD: Optical density; HBSS: Hanks balance salt solution; SD: Standard deviations; L2-L3: 2nd and 3rd-stage larvae; SPF: Specific pathogen free; ORF: Open reading frame; IgG,: Immunoglobulin E; IgG1: Immunoglobulin G class 1; IgG2a: Immunoglobulin G class 2a; IgM: Immunoglobulin M; IgE: Immunoglobulin E; IL-2: IL-4, IL-10, interleukin 2, 4, and 5, respectively; IFN-γ: Interferon γ; Th1 and Th2: T cell helper type 1 and 2; RPS: Relative percent of survival; ANOVA: Analysis of variance; LSD: Least significant difference; NS: Not significant.

## Competing interests

The authors declare that they have no competing interests.

## Authors’ contributions

YX and SJC participated in the design of the study, performed the experiments, collected and analyzed data, and completed manuscript preparation. YBY ZHZ and HY carried out animal immunoprotective trials and immunoassays. XBG, XN and XZ participated in the recombinant vaccine preparation and assisted in the migrating larvae collection from experimental animals. DSL, CDW, SXW and XRP contributed with the coordination of immunoprotective trials and participated in the design, coordination and analyses of the study. GYY conceived of the study, and participated in its design and coordination and helped to interpret the results and edited the manuscript. All authors read and approved the final manuscript.
